# Psychological distress, cardiometabolic diseases and musculoskeletal pain: A cross-sectional, population-based study of syndemic ill health in a Dutch fishing village

**DOI:** 10.7189/jogh.11.04029

**Published:** 2021-04-17

**Authors:** M Nienke Slagboom, Ria Reis, Alexander C Tsai, Frederike L Büchner, D J Annemarie van Dijk, Mathilde R Crone

**Affiliations:** 1 Public Health and Primary Care, Leiden University Medical Centre, Leiden, the Netherlands; 2 Department of Anthropology, University of Amsterdam, Amsterdam, the Netherlands; 3 The Children’s Institute, School of Child and Adolescent Health, University of Cape Town, Cape Town, South Africa; 4 Center for Global Health and Mongan Institute, Harvard Medical School and Massachusetts General Hospital, Boston, Massachusetts, USA; 5 Mbarara University of Science and Technology, Mbarara, Uganda; 6 Gemeentelijke Gezondheidsdienst Hollands Midden, Leiden, the Netherlands

## Abstract

**Background:**

Disease clustering is a growing public health concern and is increasingly linked to adverse socioeconomic conditions. Few population-based studies have focussed on interaction between non-communicable diseases. In this cross-sectional study, we examine clustering of, and synergistic interactions between, frequently occurring non-communicable diseases in Katwijk, a former fishing village in the Netherlands. Additionally, our study identifies contextual variables associated with these clusters of non-communicable diseases.

**Methods:**

In a survey among adults (>19 years) living in the former fishing village Katwijk, Netherlands, were asked about non-communicable diseases, psychological distress, self-rated health scores and contextual factors, eg, socio-demographic, psychosocial and health behavior characteristics. Interaction was measured on the additive and the multiplicative scale. We used generalized ordered logistic regression analysis to examine associations with contextual variables.

**Results:**

Three disease clusters were found to be most prevalent among the study participants (n = 1408). Each cluster involved a combination of frequently occurring conditions in this population: psychological distress (n = 261, 19%), cardiometabolic diseases (n = 449, 32%) and musculoskeletal pain (n = 462, 33%). These three diseases interact synergistically on the additive scale to increase the odds of reporting a low self-rated health. None of the disease clusters showed a statistically significant positive interaction on a multiplicative scale. Multiple contextual factors were associated with these disease clusters, including gender, loneliness, experiencing financial stress, and a BMI≥30.

**Conclusion:**

Our findings imply that psychological distress, cardiometabolic diseases and musculoskeletal pain synergistically interact, leading to a much lower self-rated health than expected. Several contextual factors are related to this interaction emphasizing the importance of a multicomponent, ecological approach.

Disease clustering is increasingly recognized as a major public health concern [1]. In the European Union alone, it is estimated that 50 million people suffer simultaneously from multiple conditions and with a rapidly aging population this number is expected to increase [2].

The field of syndemics looks at the clustering and interaction of multiple diseases, with particular attention to macro and microsocial factors that contribute to disease clustering within a population and a given context [3]. The theory posits that these intertwined health problems produce a stronger and more intense overall adverse health outcome than if each of the conditions were experienced separately [3]. With syndemic theory Singer elaborated an epidemiological framework that would allow room to describe complex health problems resulting from the interaction between epidemic diseases and harmful endemic social conditions [3,4]. This framework was introduced in the midst of long standing and well documented debates on the single disease framework, comorbidity and multimorbidity and its determinants [2,4-6].

Non-communicable diseases account for the greatest burden of disease and highest number of deaths, and disability in high income settings and are rapidly rising in low- income settings [7]. Several studies have examined the clustering of non-communicable diseases [8,9], but fewer have studied the interactions between these diseases [10,11]. As disease interaction has been theorized as one of the defining characteristics of syndemics [3], this paper examines if the presence of two or more diseases leads to a higher burden of disease than expected based on the independent contributions of the diseases considered in isolation [12,13].

This study examines these research questions in the population of Katwijk, the Netherlands. The objectives of the study were to estimate the prevalence and co-occurrence of non-communicable diseases in Katwijk, to estimate whether disease interaction contributes to self-rated health, and to identify which contextual variables were associated with the interacting clusters of non-communicable diseases.

## METHODS

### Study population

This study was set in Katwijk, the Netherlands. This Dutch former fishing village was previously known for its close-knit families, limited in-migration, social stratification, religious traditions and migratory work among men [14]. The community has experienced rapid contextual changes over the past five decades due to welfare reforms, climate change and globalization [15]. Currently, the population of Katwijk is characterized by a high prevalence of cardiometabolic diseases [16].

### Study sample and design

This study is based on secondary analysis of anonymized and pooled data from the Health Monitor Survey (2009 and 2012) [17,18] for the working age (19-64 years) and elderly age population (>65 years) in the Netherlands. This cross-sectional, population-based health survey is developed and routinely carried out every four years in all Dutch municipalities to monitor well-being and health across the general population of adults, under auspices of the Municipal Health Organization for Preventive Healthcare (GGD) [17], in collaboration with the National Institute for Public Health and the Environment (RIVM) [18] and Statistics Netherlands (CBS) [19].

To be able to compare health outcomes, the Municipal Health Organization for Preventive Healthcare (GGD) [17] draws random samples of 3%-4% of each Dutch municipality every four years [20]. The sample size for the Health Monitor Survey is calculated using the following formula [21]:

384 ÷ (1 + (383 ÷ population size target group) × 1 ÷ expected response rate).

Based on these calculations, a sample size between 700 and 750 was needed in both survey years.

A total of 1624 (2009) and 1849 (2012) people were invited to participate via a postal mailing to their home address (Figure S1 in the **Online Supplementary Document**). Individuals living in institutions (asylums, prisons or care facilities for elderly, mental health or learning disability) were excluded from participation. The data were collected through paper and pencil and online questionnaires. The working age group initially received a login code for the online questionnaire (2009 and 2012). A reminder letter and paper version were sent after two weeks (2009, 2012) and a reminder letter after four weeks of non-response (2009, 2012). The elderly population was invited to fill out a paper (2009, 2012) or online questionnaire (2009, 2012). Confidentially was explained by outlining procedures that warranted anonymous processing of data, such as assigning each respondent a unique code. Because this study is based on secondary analysis of anonymized data, ethical approval was not needed.

For our study on disease clustering and interaction, we excluded individuals that did not complete all questions on non-communicable diseases (Figure S1 in the **Online Supplementary Document**).

### Measurements

The Health Monitor Survey in 2009 and 2012 elicited information about illnesses, health and health behaviors. While the age specific questionnaires contained different questions in each wave (2009 and 2012), all questionnaires registered Self-rated health (SRH) and indicators for the presence of 17 non-communicable diseases.

Self-rated health (SRH) was used as the outcome measure and as an indicator for burden of disease. SRH has been widely acknowledged to provide an integrative summary of one’s health status and to predict morbidity and mortality [22,23].

For all non-communicable conditions, except psychological distress, prevalence of a condition was defined by the participant’s self-report of a diagnosed or undiagnosed condition within the past 12 months. Disease clustering was defined as the co-occurrence of two or more non-communicable diseases. To ensure replicability and comparability [24,25], we included HMS disease data that was available for the working age as well as the elderly age and we did not restrict on eligibility of conditions.

The following measures of self-rated health, non-communicable diseases and context were included in the analysis (**Table 1**):

**Table 1:** Characteristics of the sample, stratified according to vs high self-reported health

	n	(n%)	Low SRH (n%)	High SRH (n%)
Total (n = 1408)	1408	100.0	17.5	82.5
**Age:**				
Working age (19-64 years)	901	64.0	9.7	90.3
Elderly age (>65 years)	507	36.0	31.4	68.6
**Gender:**				
Female	761	54.5	17.9	82.1
Male	635	45.5	16.5	83.5
**Education:**				
Low	761	54.7	23.8	76.2
Middle	400	28.8	9.0	91.0
High	229	16.5	10.9	89.1
**Marital status:**				
Married or partnered	1090	77.5	15.9	84.1
Widowed or divorced	156	11.1	32.7	67.3
Single	160	11.4	13.1	86.9
**Conditions:**				
Musculoskeletal pain	462	32.9	32.3	67.7
Severe or chronic back disorder				
Severe or chronic neck and shoulder pain				
Severe or chronic pain in wrist/hand/elbow				
Rheumatoid arthritis				
Arthritis of hip or knee				
Cardiometabolic diseases	449	31.9	33.0	67.0
Coronary heart disease				
Heart failure				
Venous disease				
Stroke				
Diabetes				
Psychological distress	261	18.5	44.1	55.9
Migraine or severe headache	163	11.6	24.5	75.5
Asthma and COPD	127	9.0	40.2	59.8
Chronic inflammatory skin diseases	96	6.8	24.0	76.0
Chronic eczema				
Psoriasis				
Cancer	48	3.4	56.3	43.8
Chronic enteritis	45	3.2	60.0	40.0

SRH – self rated health, COPD – chronic obstructive pulmonary disease

#### Self-rated health

SRH was measured by a single question from the validated Short-Form 36 [26]: *‘*“In general, how would you say your health is?” (scale 1-5). 2009: ‘1 = Excellent’; ‘2 = Very good’; ‘3 = Good’, ‘4 = Fair’ or ‘5 = Poor’; 2012: ‘1 = Very good’; ‘2 = Good’; ‘3 = Fair’, ‘4 = Poor’ and ‘5 = Very poor’. Responses were dichotomized into “High SRH” (excellent/ very good/ good’) and “Low SRH” (Fair/ Poor/ Very poor) [26,27].

#### Psychological distress

Within the HMS, the presence of psychological distress (in the previous month) was assessed through the self-administered 10-item Kessler Psychological Distress Scale (K10) [28,29], a validated instrument [30] to screen for depression and anxiety in the general population. The items of the survey asses symptoms that represent the entire range of psychological distress: ‘In the past 30 days, how often have you’ (1 = none of the time to 5 = all of the time). Responses were summed, and scores ranged from 10 (no distress) to 50 (severe distress). Following previous (Dutch) population studies [30-32], a cut off score of >19 was used to categorize the respondents as having “Medium to high risk for mental health problems”.

#### *Non-communicable disease*s

Seventeen non-communicable diseases were assessed with questions developed under auspices of Statistic Netherlands [33], which have been used in health surveys in the Netherlands over the past two decades [27,34,35]. Respondents were asked to indicate, for each of the conditions separately, whether they suffered from the condition within the last 12 months (**Table 1**) [35]. A detailed description of the way the Health Monitor Survey assessed the seventeen diseases per survey wave is provided in Appendix S2 in the **Online Supplementary Document**.

Following previous population studies [36], we grouped diseases together into three system groups: “cardiometabolic disease” (heart failure/coronary heart disease/high blood pressure/venous disease/stroke and diabetes); “musculoskeletal pain” (severe and chronic back/neck and shoulder pain/chronic pain in wrist/hand/arthritis of hip or knee and rheumatoid arthritis); “chronic inflammatory skin disease” (eczema/psoriasis).

#### Context

Across survey waves, the HMS registered the following eleven variables on context:

1. *Age*: “20-34”;”35-49”; “50-64”; “65-79”; “>80”
2. *Gender*: ‘Male’; ‘Female’
3. *Education* was measured by eliciting the highest level of completed education and then grouped into three categories: “High” (university and higher professional education)’; “Middle” (pre-university and senior general secondary education); “Low” (no education, primary school, lower secondary school, pre-vocational secondary school).
4. *Civil status* was grouped into three categories “Married or partnered”; “Widowed or divorced”; “Single”.
5. *Employment status* for working age individuals (19-64 years) was assessed by asking “Which situation is most applicable to your situation?” ‘Employed’; ‘Paid work for >32 hours’; ‘Paid work for <20-<32 hours’; ‘Paid work for >12 - < 20 hours’, ‘Paid work for <12 hours’; ‘Retired’; ‘Unemployed’; ‘Not able to work and on benefits’; ‘On benefits’; ‘Full time homemaker’; ‘Student’. Elderly age individuals (≥65 years) answered the question “Is the AOW (Dutch state pension) your only source of income?” ‘Yes’; ‘No’. Employment status was then grouped into four categories “Homemaker”; “Retired”; “Benefits”; “Paid work”.
6. *Financial stress* was measured by asking questions about debt (‘No debt’; ‘Risky debt’, ‘Problematic debt, in need of help’) and experiencing difficulties in getting by financially (‘Yes’; ‘No’). Individuals reporting debt or troubles getting by financially were assigned a value of 1 for presence of financial stress.
7. *Loneliness* was assessed using the self-administered 11-item De Jong Gierveld Loneliness Scale [37,38]. Participants were asked to indicate the extent to which five positively and six negatively and formulated statements applied to their current situation, using three response categories ‘No’; ‘More or less’ ‘Yes’. The calculation of item scores is described in detail elsewhere [37,39]. Total scores could range from 0 (not lonely) to 11 (extreme lonely). A cut off score of >3 is considered to be an indication of “Medium to severe loneliness” [37].
8. *Alcohol intake* was measured based on reported number of glasses of alcoholic beverage consumed weekly. Following Dutch Health Council Guidelines 2015 [40], the GGD [17] used ≥7 (female) or ≥14 (male) drinks per week as an indicator for heavy drinking.
9. *Smoking* was assessed by the question “Do you (ever) smoke?” ‘Yes’; ‘I used to smoke’; ‘No’.
10. *Body mass index* (BMI) was calculated based on self-reported weight and height (kg/m^2^) [41] and then categorized into three standardized categories of weight “Healthy weight” (BMI<25); “Overweight” (BMI=25-29.9”; “Obesity” (BMI≥30) [41,42].
11. *Physical activity*: Participants were asked to indicate on how many days they had been physically active for at least 30 minutes, in the past week. Following the Dutch norm for physical activity for adults [43], responses (0-7 days) were grouped into ‘>5 days per week’ and ‘≤5 days per week’.

### Statistical analyses

The prevalence for each disease and accompanying self-rated health (0 = high SRH and 1 = low SRH) was calculated. This was repeated for each possible disease cluster. We fitted logistic regression models to assess for synergistic interaction between disease clusters affecting ≥4% of the population (cardiometabolic diseases [CMD], psychological distress [PD], and musculoskeletal pain [MUS]), adjusting for gender and age. Synergistic interaction was measured on both the additive and multiplicative scales. Building on previous syndemic research [44] and recent methodological recommendations [10,11], additive interaction was calculated using relative excess risk due to interaction (RERI) [45]. RERI measures whether the extent to which the effect of two exposures combined exceeds the effect of each exposure considered individually. RERI can range from - infinity to + infinity [46], with a RERI of 0 indicating no departure from additivity and a RERI of one or higher indicating a positive departure from additivity. Multiplicative interaction (M) indicates “the extent to which, on the risk ratio scale, the effect of both exposures together exceeds the product of the effects of the two exposures considered separately” [47].

Lastly, for the disease clusters with statistically significant additive or multiplicative synergistic interaction, we performed logistic regression analysis to examine associations with contextual variable using an ordered outcome (no disease, one disease, two diseases) [48]. Due to violations of the assumption of proportional odds, we used partial proportional odds regression to estimate associations between this ordered outcome variable and the contextual variables described previously [49]. The partial proportional odds regression model is similar to the ordered logistic regression model except that it permits selected regression coefficients associated with covariates to differ across the logit equations. We constrained the regression coefficients to be equal across the logit equations except for explanatory variables where the proportional odds assumption was violated. For these covariates, we report two odds ratios: one odds ratio that estimates the association between the explanatory variable and the probability of having one or two diseases (compared with no disease), and one odds ratio that estimates the association between the explanatory variable and the probability of having two diseases (compared with one disease or no diseases). Statistical analyses were performed using SPSS version 25 (IBM Corp., Armonk, NY). R Studio 1.3.959 (RStudio PBC, Boston, MA, USA) was used for the interaction assessment [50]. Stata (StataCorp., College Station, TX, USA) was used for the partial proportional odds regression analysis [48].

## RESULTS

The initial Health Monitor Survey pooled sample consisted of 1699 participants: 817 respondents from 2009 and 882 from 2012 (Figure S1 in the **Online Supplementary Document**). The analysis for this syndemics study was conducted based on a sample of 1408 participants (**Table 1**).

### Clustering

Thirty four percent of the population reported two or more non-communicable diseases. **Table 2** shows that the three most prevalent clusters were musculoskeletal pain and cardiometabolic diseases (15%), musculoskeletal pain and psychological distress (9%) and cardiometabolic diseases and psychological distress (7%).

**Table 2:** Logistic regression models estimates of the additive and multiplicative interactions between disease pairs in their associations with self-rated health, adjusted for age and gender

Condition 1	Condition 2	%n	Low SRH %n	AOR (95%CI)	RERI (95%CI)	Multiplicative interaction
**Cardiometabolic diseases**	**Psychological distress**					
0	0	56.7	6.8	1		
1	0	24.8	22.1	3.9 (2.6, 5.65)		
0	1	11.4	27.3	4.7 (3.05, 7.51)		
1	1	7.1	71.0	32.52 (19.44, 54.38)	25.04 (9.18, 40.89)	1.73 (0.88, 3.40)
Musculoskeletal pain	Psychological distress					
0	0	57.5	7.3	1		
1	0	24.0	21.3	3.51 (2.42, 5.10)		
0	1	9.7	27.7	4.92 (3.09, 7.84)		
1	1	8.8	62.1	20.68 (13.06, 32.76)	10.40 (2.81, 17.99)	1.20 (0.61, 2.35)
Cardiometabolic diseases	Musculoskeletal pain					
0	0	50.4	6.6	1		
1	0	16.8	21.2	3.84 (2.47, 5.93)		
0	1	17.7	20.5	3.56 (2.31, 5.49)		
1	1	15.1	46.0	12.04 (8.04, 18.04)	5.64 (1.73, 9.55)	0.88 (0.48, 1.60)

AOR – adjusted odds ratio, CI – confidence interval, SRH – self rated health, RERI – relative excess risk due to interaction

### Disease-disease interaction

**Table 3** presents the adjusted odds ratios (AORs) for lower self-rated health corresponding to the pairwise association between the three most frequently occurring disease clusters. The RERI exceeded 1 for each of these three disease clusters, indicating positive departures from additivity, ie, that the diseases have an interactive association with low self-rated health that is greater than what would be expected on the additive scale. None of these disease clusters showed statistically significant positive interaction on the multiplicative scale.

**Table 3:** Contextual factors associated with the different disease clusters using partial proportional odds regression

		CMD-PD					MUS-PD				CMD-MUS			
		**1 or 2 diseases vs none**		2 diseases vs no diseases or 1 disease*			**1 or 2 diseases vs none**		2 diseases vs no diseases or 1 disease*		**1 or 2 diseases vs none**		2 diseases vs no diseases or 1 disease*	
**Variable**		**AOR**	**95%CI**		**AOR**	**95%CI**	**AOR**	**95%CI**	**AOR**	**95%CI**	**AOR**	**95%CI**	**AOR**	**95%CI**	
Age (years)	19-34#	Ref					1				1				
	35-49	0.90	(0.57,1.41)				0.99	(0.65, 1.52)			1.65	(1.03, 2.65)			
	50-64	2.17	(1.37, 3.44)				1.69	(1.08, 2.65)			5.35	(3.29, 8.70)	10.70	(5.29, 21.62)	
	65-79	3.52	(1.81, 6.84)				2.55	(1.32, 4.93)	0.97	(0.44,2.14)	8.13	(4.15, 15.92)	17.05	(7.53, 39.06)	
	>80	6.59	(2.73, 15.91)				2.75	(1.19, 6.36)			25.64	(10.16, 64.66)			
Gender	Male#	Ref					1				1				
	Female	1.67	(1.28, 2.19)				1.85	(1.43, 2.41)			1.46	(1.12, 1.91)			
Education	High#	Ref					1				1				
	Middle	0.98	(0.67, 1.44)				1.14	(0.78, 1.65)			1.17	(0.81, 1.70)			
	Low	1.20	(0.84, 1.72)				1.11	(0.78, 1.58)			1.14	(0.81, 1.62)			
Civil status	Married or partnered#	Ref					1				1				
	Widowed or divorced	1.19	(0.80, 1.77)				1.11	(0.75, 1.65)			1.01	(0.69, 1.48)			
	Single	1.37	(0.89, 2.10)				0.95	(0.62, 1.44)			0.96	(0.61, 1.50)			
Employment	Paid work#	Ref					1				1				
	Retirement	1.51	(0.89, 2.57)				1.20	(0.71, 2.02)	4.50	(2.13, 9.49)	1.39	(0.84, 2.30)			
	Benefits	1.94	(1.03, 3.65)				1.49	(0.77, 2.87)	5.40	(2.31, 12.63)	1.36	(0.70, 2.62)			
	Homemaker	1.13	(0.72, 1.79)				1.11	(0.70, 1.75)	3.38	(1.66, 6.85)	1.31	(0.85, 2.02)			
Financial stress	No#	Ref					1				1				
	Yes	1.91	(1.38, 2.66)				1.90	(1.38, 2.62)			1.12	(0.78, 1.61)	1.85	(1.17, 2.91)	
Loneliness	Low score#	Ref					1				1				
	High score	1.82	(1.39, 2.37)		3.50	(2.16, 5.66)	2.22	(1.73, 2.84)			1.13	(0.88, 1.45)			
Smoking	No#	Ref					1				1				
	Former smoker	1.06	(0.79, 1.40)				1.07	(0.82, 1.42)			1.17	(0.87, 1.58)	0.75	(0.52, 1.08)	
	Yes	1.14	(0.81, 1.61)				1.19	(0.85, 1.65)			1.08	(0.77, 1.52)	0.86	(0.58, 1.28)	
Alcohol intake	<7(f)/14(m) units/week #	Ref					1				1				
	≥7(f)/14(m) units/week	1.20	(0.89, 1.61)				0.79	(0.59, 1.04)			1.30	(0.95, 1.77)			
Body mass index	BMI<25#	Ref					1				1				
	BMI 25-29.9	1.46	(1.10, 1.94)				1.21	(0.92, 1.59)			1.75	(1.33, 2.31)			
	BMI≥30	3.05	(2.16, 4.32)				1.62	(1.15, 2.27)			3.54	(2.52, 4.98)			
Physical activity	>5 days/week#	Ref					1				1					
	≥5 days/week	1.35	(1.05, 1.73)				1.07	(0.83, 0.36)	1.69	(1.09, 2.62)	1.24	(0.97, 1.57)				

CMD – cardiometabolic diseases, MUS – musculoskeletal pain, PD – psychological distress, AOR – adjusted odds ratio, CI – confidence interval, # – reference group, m – male, f – female

*Two odds ratios are reported for covariates where the associated estimates vary across the logit equations; one odds ratio is reported for covariates where the proportional odds assumption was not violated.

### Disease-context association

In the analysis of correlates of disease clustering (Table S3 in the **Online Supplementary Document**), most of the explanatory variables did not violate the proportional odds assumption, meaning that the association between the explanatory variable and moving from a lower disease cluster category (from no diseases to 1 disease) to a higher disease cluster category (from no diseases or 1 disease, to 2 diseases) was similar irrespective of the level of the dependent variable. Across the three disease clusters, having a greater number of diseases was associated with being of middle age and older, gender (female), financial stress, and body weight (BMI>30).

For a few explanatory variables, violations of the proportional odds assumption were noted. For example, in the analysis of the cardiometabolic disease-psychological distress disease cluster, the effect of loneliness differed across the logit equations, where the effect was much larger for moving from no diseases vs 1 or 2 diseases compared with the effect of moving from no diseases or 1 disease vs 2 diseases.

## DISCUSSION

In this cross-sectional, population-based syndemics study, we examined the clustering of and synergistic interactions between frequently occurring non-communicable conditions among adults in a Dutch former fishing village. Three disease clusters were found to be most prevalent in this village, involving combinations of psychological distress, cardiometabolic diseases and musculoskeletal pain. We showed that the three diseases interact in mutually exacerbating ways, meaning that these combinations of non-communicable diseases lead to a much lower self-rated health than would be expected based on their independent contributions to self-rated health. We also showed that these three disease clusters were not only associated with age; our findings indicate that they were also more likely to occur among people, particularly women, whose health is impacted by financial stress and increased body weight. Lastly, people suffering from psychological distress in combination with either cardiometabolic disease or musculoskeletal pain were more often not engaged in paid work, suffered more from loneliness, and scored low on physical activity.

Overall, our findings add to the body of knowledge on depression and diabetes syndemics among populations that experienced social and economic hardship [8,51-53]. Our study provides a unique empirical test of biological-biological and biological-social relationships of non-communicable conditions in the general population. While the underlying pathophysiological mechanisms for synergistic interaction could not be determined through this study, previous studies have argued that interaction between depression, cardiometabolic conditions, and musculoskeletal pain is most likely associated with a systemic inflammatory dysregulation [54-57], which has been linked to stress, possibly from the prenatal phase onwards [58], and is believed to destabilize the autonomic nervous system and dysregulate immune response [59,60]. Building on fishermen health studies [61,62], Slagboom [15] argued that communities like Katwijk are likely vulnerable to such adverse disease interactions “because of their history of harsh working conditions, occupational hazards, as well as the adverse socioeconomic conditions in which fishermen communities typically lived, characterized by income uncertainty and poor access to health care”. In a previous syndemics study in Katwijk [15], following the identification of psychological distress, cardiometabolic conditions and musculoskeletal pain, life events and contextual factors were examined more closely, using a life-course approach and qualitative methodology. This study showed that people suffering from these diseases often reported a history of adverse life events beginning in early childhood and that these diseases often restricted the ability to work - a major stressor in a context with a distinct work ethic and sociocultural norms that emphasize perseverance and being strong. Distress over ill health and income was often experienced during home confinement, which might explain the associations with loneliness, limited physical activity and financial stress as reported in this present study.

Direct comparison of our findings with other population-based studies proved to be complex, as most studies in this literature have examined multimorbidity and have focused on older-age populations or clinical samples [24]. The strong impairing effect of psychological distress combined with cardiometabolic diseases or pain found in this study, however, is consistent with previous studies [63,64]. A worldwide study in the Lancet, for example, showed that depression combined with other health conditions “incrementally worsens health outcomes compared with depression alone, with any of the chronic diseases alone, and with any combination of chronic diseases without depression” [65]. Interestingly, our study showed that synergistic interaction was not confined to the presence of psychological distress [66]. The presence of musculoskeletal pain too, increased the odds of poor self-rated health incrementally.

Our findings on contextual factors help to reconcile key findings in (social) epidemiology, which have shown that multimorbidity is a multifactorial phenomenon that is not merely related to elderly age [67-69]. Within the field of syndemics, these findings confirm the link between an early onset of disease clustering, socio economic position and gender as described in previous qualitative studies of depression and diabetes [8,53]. In line with another study of disease clustering in a welfare state setting, our findings point at heterogeneity in contextual factors [70], which are partly dependent on the disease cluster concerned.

### Limitations

Limited causal conclusions can be drawn from this study given its use of cross-sectional self-report survey data. Self-report of presence and history of conditions has been questioned for accuracy of estimating (true) prevalence of diseases and critiqued for limiting comparability with other studies. Further, the study results could be biased due to voluntary response, oversampling of the working age population and combining of multiple data sets.

While more research is certainly needed to verify the reported patterns, the measurement of conditions, including psychological distress, might be quite accurate and useful for syndemic research in contexts like Katwijk where help seeking is often delayed and underreporting of health conditions is common [15].

Because of the cross-sectional design, we could not draw inferences about causality, directionality or temporality of the contextual factors. As such, we could not fully explain how these factors act and why contextual factors act differently depending on specific disease combinations. No evidence for an association, however, does not necessarily imply the absence of a relation, this is especially true for factors which are well documented in studies of fishermen health and could have contributed to the development of disease earlier in life, such as heavy drinking and smoking. While our findings indicate that it is possible to test the three tennets of syndemic theory using routine data, our findings underscore the need for more longitudinal and mixed methods research, including more refined measurements of context, to come to a better understanding of adverse disease interaction on a population level.

## CONCLUSION

Psychological distress, cardiometabolic diseases and musculoskeletal pain were found to interact in mutually exacerbating ways, leading to a much lower self-rated health than expected. Adverse disease interaction between these conditions is likely to be shaped by multiple social conditions, including gender, financial stress and loneliness.

Our findings suggest that musculoskeletal pain is a useful focus for future syndemics research, for example in other populations with a strong history or shift to “blue collar” occupations that do not require a college degree, in which a high prevalence of chronic pain, opioid medication treatment, disability, and substance use disorders has been documented [71-73]. Such research needs to incorporate a historical and placed based approach, amongst others focusing on working conditions and power relations.

The social interconnectedness of diseases and context as shown in this study emphasize the need to analyse pathways to non-communicable or ‘lifestyle related’ diseases outside a discourse of “responsibilization” [74,75]. Our findings support that syndemic vulnerability is unlikely to be fully addressed with approaches such as medical screenings and treatments or public health interventions that target individual behavior change [76]. Instead, a multicomponent, ecological approach is needed, which integrates interventions directed at different domains and educates policymakers and care professionals about the social interconnectedness of psychosocial well-being, cardiometabolic and painful conditions.

## Additional material

Online Supplementary Document
