# Supplementary material for: Psychological distress, cardiometabolic diseases and musculoskeletal pain: A cross-sectional, population-based study of syndemic ill health in a Dutch fishing village
Source: J Glob Health. 2021 Apr 17;11:04029. doi: 10.7189/jogh.11.04029 (PMC8068410; doi:10.7189/jogh.11.04029)
Supplement: Online Supplementary Document [file jogh-11-04029-s001.pdf]

Supplementary 1

Figure 1. Flowchart Participant inclusion

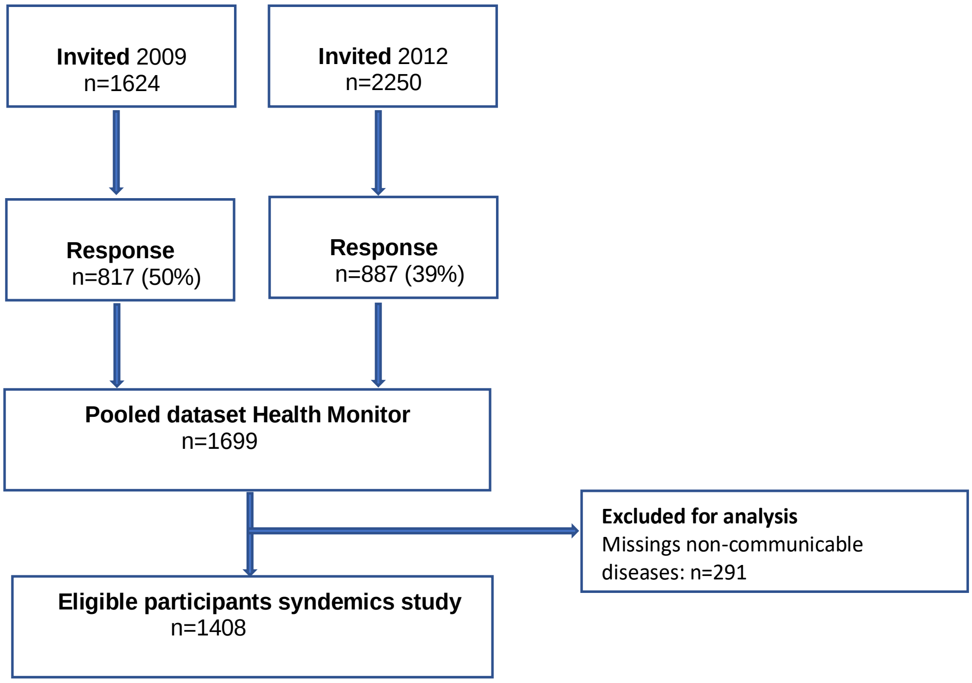

Appendix S2 Variable description non-communicable diseases

The following seventeen diseases were assessed across the two age groups and two waves of the Health Monitor Survey (2009;2012):

1) Severe or persistent chronic back disorder; 2) severe or chronic neck and shoulder pain; 3) severe or chronic pain in wrist/hand/elbow'; 4) arthritis of hip or knee; 5) 'rheumatoid arthritis'; 6) chronic eczema; 7) psoriasis; 8) asthma, chronic bronchitis, emphysema or COPD; 9) chronic enteritis (> 3 months); 10) migraine or severe headache; 11) venous condition; 12) high blood pressure; 13) coronary heart disease 14) stroke; 15) cancer; 16) heart failure 17) diabetes

In 2009 the conditions were assessed through the following question: "Based on the following list of illnesses and conditions, please tick if you currently have or have had these in the last 12 months": 'Yes, not determined by a doctor'; 'Yes, determined by a doctor'; 'No'. (variable 1-17)

In 2012 the conditions were measured in the following ways: "Please tick if you currently have or have had these in the last 12 months": 'Yes'; 'No' (variable 2-12) or "Have you ever had…" (variable 13-15), followed by "If so, have you had this [disease, red.] in the past 12 months?" 'Yes'; 'No'. Heart failure (variable 16) was measured by asking "Have you had another [other than cororary heart disease, red]. severe heart condition in the past 12 months, for example heart failure or agina pectoris?" 'Yes'; 'No'. Diabetes (variable 17) was assessed by asking "Have you been under treatment or monitored by the general practitioner or specialist in the past 12 months" or "Did you start using insulin within 6 months of your diabetes diagnosis?" 'Yes'; 'No'.

Table S3 Percentages contextual factors

Contextual factors	Total n=1408	No CMD, No PD n=798	CMD or PD n=510	CMD and PD n=100	No Mus, No PD n=809	Mus or PD n=475	Mus and PD n=124	No Mus, No CMD n=710	Mus or CMD n=485	Mus and CMD n=213
Age										
19-34, %n	17	75	23	3	71	26	3	84	16	1
35-49	22	75	24	1	70	26	5	72	25	3
50-64	25	55	39	6	55	36	9	42	41	17
65-79	31	39	49	12	47	42	13	30	44	27
>80	5	32	42	26	38	35	28	16	48	36
Gender										
Male, %n	46	61	33	6	64	30	6	53	34	14
Female	55	53	39	8	52	37	10	48	35	16
Education										
Low, %n	55	49	41	10	52	36	12	42	37	21
Middle	29	66	30	4	63	32	6	62	29	9
High	17	67	29	4	67	29	4	61	32	7
Civil status										
Married or partnered, %n	78	59	34	6	59	34	8	51	35	15
Widowed or divorced	11	36	47	17	40	39	21	29	46	26
Single	11	61	35	4	66	30	4	71	23	7
Employment										
Paid work, %n	51	71	28	2	67	30	3	68	26	6
Housewife/man	10	54	38	8	51	36	13	40	42	18
Benefits	4	43	43	14	47	33	20	49	35	16
Retirement	36	39	48	13	47	39	14	29	44	27
Financial stress										
No, %n	84	59	35	6	60	33	8	50	35	15
Yes	16	47	41	12	47	38	15	54	29	17
Loneliness										
Low score, %n	65	63	33	3	66	30	5	54	34	13
High score	35	45	42	14	43	41	16	46	36	19
Smoking										
No, %n	39	61	32	8	61	31	9	55	30	15
Former smoker	40	51	41	8	55	37	9	42	41	17
Yes	21	60	35	5	58	34	8	60	29	12
Alcohol intake										
<7(f)/14(m) units/wk, %n	78	57	36	7	59	32	9	51	36	14
≥7(f)/14(m) units/wk	22	59	35	7	52	40	8	52	31	18
Body mass index										
BMI <25, %n	39	67	28	5	63	29	8	65	27	8
BMI 25-29.9	43	56	37	7	57	35	8	46	39	15
BMI ≥30	18	36	52	12	46	40	14	31	39	30
Physical activity norm										
> 5 days/wk, %n	43	58	37	5	57	36	3	51	36	13
≥ 5 days/wk	57	56	36	9	58	32	6	51	32	17

CMD - cardiometabolic diseases; MUS - musculoskeletal pain; PD - psychological distress
